# Experiences of young people growing up in a family with Huntington's disease: A meta‐ethnography of qualitative research

**DOI:** 10.1002/jgc4.1886

**Published:** 2024-03-12

**Authors:** Hollie Cooper, Jane Simpson, Maria Dale, Fiona J. R. Eccles

**Affiliations:** ^1^ Division of Health Research Lancaster University Lancaster UK; ^2^ Leicestershire Partnership NHS Trust Leicestershire UK

**Keywords:** family, Huntington's disease, lived experience, meta‐ethnography, qualitative, young people

## Abstract

Huntington's disease is a genetic neurodegenerative condition with wide physical and psychological impacts. Children of a parent with the condition have a 50% chance of carrying the gene expansion and developing the condition themselves. This systematic review and meta‐ethnography presents a synthesis of the qualitative research on the experiences of young people growing up in a family with Huntington's disease. The MEDLINE, PsycINFO, and CINAHL databases were systematically searched, and 13 papers met the inclusion criteria. Through the process of meta‐ethnography, four themes were identified highlighting aspects of childhood that were stolen and fought for: thief of relationships, thief of self, thief of transparency, and search for reclamation. Within the themes, the complex challenges young people faced when growing up in a HD family were explored such as the impact of adverse childhood experiences and the possible effects of HD on attachment and social relationships. Clinical implications are considered, and recommendations are made for future research.

**What is known about this topic**

Young people often become carers of a parent and are required to fulfill typical adult roles when living in a HD family. No review has been conducted to date about the experiences of young people growing up in a family affected by Huntington's disease.

**What this paper adds to the topic**

This review synthesizes, from 13 qualitative studies, the experience of young people growing up in a family affected by HD. It explores the complexities young people face, this impact on their lives and highlights the difficulties young people have in accessing support.

## 1 INTRODUCTION

Huntington's disease (HD) is a genetic condition in which psychological distress is common. Due to its genetic nature, a child of an individual with the expanded HD gene will have a 50% chance of inheriting and subsequently developing the condition. HD is incurable and progressive, with increasing problems with movement and cognitive impairment leading to dementia. The global prevalence is approximately 2.7 per 100,000 (Pringsheim et al., 2012) but is higher in some countries, for example, 10 per 100,000 in the United Kingdom (Furby et al., 2022).

Predictive genetic testing can be undertaken to determine whether an individual carries the expanded gene and guidelines recommend that this should occur at age 18 or older, barring exceptional circumstances.

Individuals are not diagnosed with HD until they display motor difficulties, for example, chorea or loss of balance (Novak & Tabrizi, 2010). Therefore, those with the gene expansion but no motor signs are known as presymptomatic or premanifest and those who have not sought genetic testing but have a biological parent (50% risk) or grandparent (25% risk) with HD are known as being at risk (Novak & Tabrizi, 2010). Usual onset of motor symptoms occurs between the ages of 30 and 50 (McColgan & Tabrizi, 2018), though with wide variation (Frich et al., 2014). Death occurs approximately 24 years after a HD motor diagnosis (Rodrigues et al., 2017). Currently, no medications slow the progression of the disease, though help with symptom control is available for some elements, such as involuntary movements (Venuto et al., 2012).

### 1.1 HD in families

In HD families, many complex challenges arise that extend past the physical and neurological effects of the disease. For example, Vamos et al. (2007) found low levels of family cohesion and verbal expression, alongside higher levels of conflict compared to non‐HD families. A qualitative study, including parent/adult child dyads, explored the impact of the condition on the family throughout HD progression, concluding that understanding the needs of families experiencing HD is required (Maxted et al., 2014).

However, one voice that needs to be amplified through research on HD within families is that of young people. It is important to understand the perspective of young people as, due to the typical age of onset of HD, parents often present with symptoms when their children are entering the life stage of becoming a young person (Driessnack et al., 2012). While some studies have highlighted the impact of HD on the family unit (Van der Meer et al., 2006; Vamos et al., 2007; Williams et al., 2009; Huniche, 2011; Sobel & Cowan, 2000), we do not as yet have a synthesis of the experiences of young people. This is despite early research indicating that 48% of children from a HD family experienced psychological distress (Folstein et al., 1983).

Consequently, the following review aims to answer the question: ‘What is the experience of young people who have grown up in a family with HD?’. Given the focus of the review question and its emphasis on lived experience only qualitative studies were included. A meta‐ethnography was chosen as this can bring new understanding (Campbell et al., 2011); it does not simply aggregate findings (Noblit et al., 1988) but can produce new evidence on experience (Campbell et al., 2011) grounded in the primary studies' data (France et al., 2019). Findings from such syntheses have the potential not only to inform future research but also the development of appropriate interventions and services (France et al., 2019).

## 2 METHOD

Conceptualization of data varies across methodologies. In this review, and according to the theoretical position of meta‐ethnography, data were perceived as both authors' interpretations and participant quotes. The synthesis was conducted following Noblit et al.'s (1988) detailed seven step guidance.

### 2.1 Identifying relevant papers

The following databases were searched—MEDLINE, CINAHL, and PsycINFO—focusing on HD and qualitative designs. An example of free text terms and subject headings can be seen in Table S1. No data restrictions were applied, and the final search was conducted in December 2022.

### 2.2 Inclusion/exclusion criteria

To be included, papers had to:

1. Include qualitative data collected via qualitative methods with participants' quotes so that beliefs and experiences could be understood.
2. Be published in a peer‐reviewed journal.
3. Focus on the experience of a contemporaneous account or include retrospective accounts of childhood by an adult who grew up in a family with HD.
4. Have data relevant to the research question easily identifiable and extractable.
5. Be in English.

Papers were excluded when they contained:

1. Comparative or dyadic studies where the experience of the young person in a HD family could not be clearly extracted or where such distinction could not be clearly made (Duncan et al., 2008; MacLeod et al., 2014; Mand et al., 2013; Stuttgen et al., 2021).
2. Mixed methods studies with no in‐depth qualitative work (Chase et al., 2022; Kavanaugh, 2014; Kavanaugh et al., 2016; Lewit‐Mendes et al., 2018; Williams et al., 2013)

### 2.3 Search results

**FIGURE 1:**
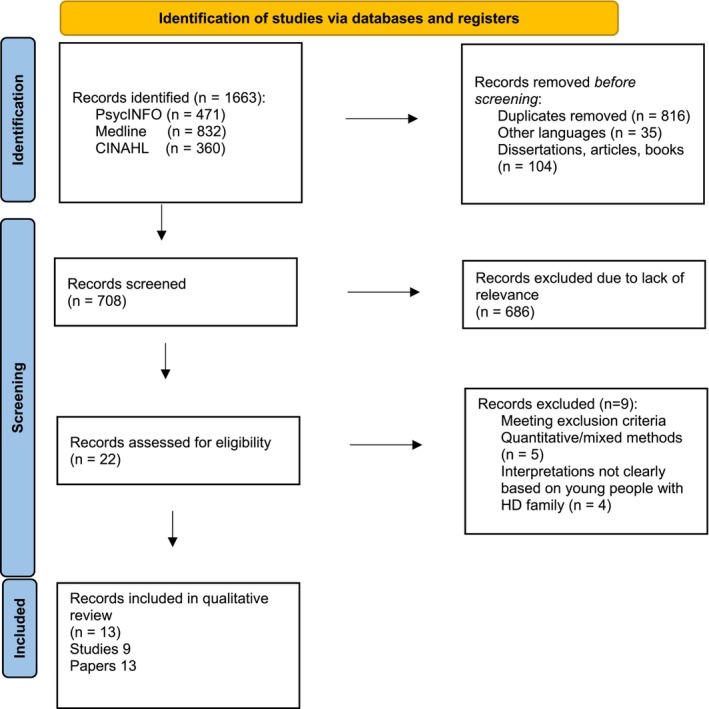
A PRISMA flow diagram to illustrate study identification via databases. From: Page et al., 2021. For more information, visit: http://www.prisma‐statement.org/.

In the initial search, 1663 articles were identified of which 816 were duplicates, 35 were not in English, and 104 were not peer reviewed and were therefore removed. The remaining 708 were appraised using the title and abstract to establish relevance. Of these, 686 were excluded due to irrelevance to the research question or not meeting the inclusion criteria. Of the final 22 papers which were read in full, 5 were excluded either because they were quantitative or mixed methods and 4 because the data on young people in HD families were not clearly extractable. This resulted in 13 papers from 9 studies being included in the review. A PRISMA diagram (Page et al., 2021) to illustrate this process can be seen in Figure 1.

### 2.4 Study characteristics

Study characteristics are shown in Table 1. Studies were carried out between 2007 and 2022 in four countries: the USA (*n* = 5), Norway (*n* = 3), Scotland (*n* = 3), and Australia (*n* = 2). The following papers used the same participant sample to address differing research aims:

- Forrest Keenan et al. (2009) and Forrest Keenan et al. (2007).
- Kjoelaas, Jensen, and Feragen (2022), Kjoelaas, Feragen, and Jensen (2022) and Kjoelaas et al. (2020).
- Williams et al. (2009) and Sparbel et al. (2008)

**TABLE 1:** Study characteristics.

Author	Title	Country	Participants	HD status	Aims	Method of data collection	Method of analysis	Findings
Dondanville et al. (2019)	‘This could be me’: exploring the impact of genetic risk for Huntington's disease young caregivers	USA	13 individuals, 15‐25 years old recruited from local youth groups/support groups 11 female 2 male	At risk	Understand the interaction between young caregivers' perception of risk, the caregiving experience and their thoughts about predictive testing	Interviews	Thematic analysis	Caregiving is affected by genetic risk which evokes feelings about the future and possible diagnosis and affects plans for testing. Knowledge for genetic counselors and support needed for young people
Duncan et al. (2007)	‘Holding your breath’: interviews with young people who have undergone predictive genetic testing for Huntington disease	Australia	15‐24 years, 8 young people who had undergone testing, 4 male 4 female	x2 gene positive, x6 gene negative	Explore the experience of predictive testing from the young person's perspective, document the impact of testing on young person's lives	Interviews	Thematic analysis	Three themes: living as though gene positive Risk behaviors, complex pasts. Themes after testing: identity, living again. All reported on testing in the wider context of growing up in a HD family. No one regretted testing
Forrest Keenan et al. (2015)	Help or hindrance: young people's experiences of predictive testing for Huntington's disease	Scotland	17‐26 years, 12 participants recruited via genetic services	All at risk at recruitment	Explore young people's experiences of predictive testing, the impact of the result and gaps in support	Interviews	Thematic analysis	Three testing experiences regardless of result: empowerment, ambivalent, poor experience. Changes in family dynamics made the post‐test stage difficult
Forrest Keenan et al. (2009)	How young people find out about their family history of Huntington's Disease	Scotland	33 Young people aged 8–28 21 female 12 male	X26 at risk X1 gene positive X5 gene negative X1 diagnosed	Explore young people's experiences of finding out about the presence of HD in the family	Interviews	Thematic analysis	Four main themes: always been told, gradually told, kept a secret, new diagnosis
Forrest Keenan et al. (2007)	Young people's experiences of growing up in a family affected by Huntington's Disease	Scotland	33 Young people aged 8–28 21 female 12 male	X26 at risk X1 gene positive X5 gene negative X1 diagnosed	To describe the experience of young people growing up in HD families	Interviews	Thematic analysis	Four main themes: young people as carers, worried well, those who cope, those in need
Gong et al. (2016)	Impact of Huntington's Disease Gene‐positive status on pre‐symptomatic young adults and recommendations for genetic counselors		15 Young people aged 20–33 12 female 3 male	All carriers	To explore how a positive result affects the attainment of milestones in education, relationships etc	Interviews	Grounded theory and thematic analysis	Three main categories: changes in attitude and approach to life, influences on milestones of adulthood, suggestions for genetic counselors – new finding that young adults have awareness that healthy years are limited
Kavanaugh et al. (2015)	‘It'd be nice if someone asked me how I was doing. Like, because I will have an answer’: Exploring support needs of young carers of a parent with Huntington's disease	USA	40 participants between 12 and 20 with a family member with HD	At risk	To understand the roles of young care givers and their support needs	Interviews	Content analysis	Themes: instrumental support, emotional support, personal needs – implications for social work and health care professionals in designing support programs
Kjoelaas, Jensen, and Feragen (2022)	‘I knew it wasn't normal, I just didn't know what to do about it’: adversity and caregiver support when growing up in a family with Huntington's Disease	Norway	36 participants aged 13–65 years old	At risk	Explore ACEs of children who grew up in HD families and understand their perception of caregiver support	Semi‐structured interviews	Interpretative Phenomenological Analysis	Participants with support tolerate adversity better than those without support who feel overwhelmed
Kjoelaas, Feragen, and Jensen (2022)	Social support experiences when growing up with a parent with Huntington's disease	Norway	36 participants aged 13–65 years old	At risk	Explore young people's experience of accessing social support outside of the parent–child relationship in HD families	Semi‐structured interviews	Thematic Analysis	Social support can help when there is a lack of support at home though there are barriers to be addressed
Kjoelaas et al. (2020)	The ripple effect: a qualitative overview of challenges when growing up in families affected by Huntington's Disease	Norway	36 young people and adults 13–65 years old	At risk	Explore the challenges of growing up in a HD family	Semi‐structured interviews	Thematic analysis	Four main themes: family functioning, emotional and reactions, social functioning, public and care services
Mand et al. (2015)	‘I'm scared of being like mum’: the experience of adolescents living in families with Huntington disease	Australia	10 young people, 13–20 years old 9 under 18 at the time, none requested a test. Purposive sample	At risk	Explore psychosocial context of young people in HD families and understand their experiences and challenges they face	Semi‐structured interview	Thematic analysis	Young people in HD families face greater responsibilities and stressors
Sparbel et al. (2008)	Experiences of teens living in the shadow of Huntington's Disease	Canada	32 young people 14–18 years old	X27 at risk X5 negative	Explore the experience of teens living in HD families	Focus groups	Content analysis	Data showed a complex and often painful family environment. Watching and waiting Living in the shadow Alone in the midst of others Family life is hard Having to be an adult
Williams et al. (2009)	Caregiving by teens for family members with Huntington's Disease	Canada	32 young people 14–18 years old	X27 at risk X5 negative	Explore the experiences of teens caring for family members with HD	Focus groups	Content analysis and narrative synthesis	Two themes consistent with adult caregiving literature: tasks and responsibilities and subjective burden. Two of these were specific to young care givers: caregiving in the context of personal risk and decisional responsibility

Thus, the 13 papers for inclusion represented data from 9 original studies. In total, data were gathered from 199 individuals who formed sample sizes of between 5 and 40 of varied HD status at the point of recruitment (160 at risk, 22 gene positive, 16 gene negative, 1 diagnosed). Participants were aged between 8 and 65 years old, resulting in current and retrospective accounts of childhood and adolescent experiences of growing up in a family with either a HD parent or close relative. Four of the studies presented a mixture of current and retrospective accounts (Gong et al., 2016; Kjoelaas et al., 2020; Kjoelaas, Feragen, & Jensen, 2022; Kjoelaas, Jensen, & Feragen, 2022). The decision to include such an age variance was informed by the wider existing literature on young carers which includes ages 5–18 (e.g., Bauman et al., 2006; Gates & Lackey, 1998) and the United Nations' (2003) definition of young people being between 15 and 24. However, research focusing on young people in this age bracket (5–24) in HD families is limited. Consequently, retrospective accounts of growing up in an HD family where the participant was over 25 and reflecting on their experience of being a young person were included.

### 2.5 Quality of the selected studies

Due to study selection in meta‐ethnography being guided by what is available (Noblit et al., 1988) and to ensure conceptually rich papers were included, studies were not excluded based on methodological quality. However, quality assessment of included studies was still considered important, although it is important to note that appraisal tools have a focus on methodological strength and not conceptual strength (Long et al., 2020; Toye et al., 2014).

To understand the quality of the included studies, the Critical Appraisal Skills Programme (CASP) checklist (Burls, 2014) was used as it has been endorsed for qualitative studies by Cochrane (a source of influential advice on the conduct of reviews) and the World Health Organization (Noyes et al., 2018). The CASP provides a framework for exploring 10 questions with answers of ‘yes’, ‘can't tell’, and ‘no’ to appraise the quality of a study. The CASP rating for each study can be seen in Table S2 and shows a relatively strong collection of papers. The CASP highlighted the main weakness across papers to be lack of reflexivity on the relationship between author and participants.

### 2.6 Analysis

Meta‐ethnography was chosen as it provides a way to present a new understanding of a phenomenon that is of a higher order, that is, it is not presenting or simply collating what is already written within the included studies (Sattar et al., 2021). Using an approach such as narrative synthesis would rely on what the studies alone offer and would not offer this additional interpretative element. Following the seven steps advocated by Noblit et al. (1988) and meta‐ethnography guidance (Sattar et al., 2021), papers were first read and re‐read with each read resulting in detailed annotation. To preserve the context and meaning across studies, original papers were revisited throughout the analysis process to ensure the original meaning had not been lost. To assist this, a table was used to record quotes from each study. This ensured that the context of what was being shared was not lost during the translation into third order interpretations. Metaphors and analogies were also used to explore how the studies related to one another without losing the context of the information offered and holistic meaning was preserved. This enabled key concepts to be drawn from each paper which were placed into a table.

Concepts were then transferred into a second table during translation demonstrating which concept appeared in which paper (Table S3). Through this process, the four third order constructs (i.e., final themes) were formed. The first author was responsible for the formation of third order constructs and discussed these regularly with the other authors. Reflexivity was addressed through the keeping of a journal alongside the formation of constructs so the author could remain aware of how the data were being interpreted and could discuss this with the other authors. The first author had no personal lived experience in the context of HD; therefore, her social and cultural beliefs may have affected interpretations, though this was minimized through the meta‐ethnographic approach. The second and last authors are academics with knowledge of HD through research, and the third author is a clinician and academic who works day to day with people with HD. All the authors are clinical psychologists. To ensure themes were rooted in the data, the analysis was regularly discussed by all three co‐authors, to discuss different interpretations. Given the expertise of the authors, it is likely psychological experiences would be prioritized and noted, with the wider knowledge of HD in the team ensuring the findings also made sense in the context of research and clinical experience.

Discussion within the authorship team throughout this process aided in maintaining the quality of the findings and methodological rigor (Atkins et al., 2008). To improve rigor, two randomly selected papers were coded by a colleague external to the research team with no knowledge of HD. Concepts generated by this individual were compared to those created by the primary author (HC) for the two papers and indicated a high level of similarity. The same colleague also completed a CASP for two papers and generated the same ratings as the primary author.

## 3 FINDINGS

Through synthesizing the 13 papers, four interrelated constructs were identified: thief of relationships, thief of self, thief of transparency, and search for reclamation. Though the process of meta‐ethnography enables reciprocal and refutational translations, no refutational translations were identified.

### 3.1 Thief of relationships

HD stole young people's relationships with their parents, family members, and friends and contributed to their lack of social support networks. Within this theme were factors that influenced the taking of relationships such as parental absence, grief and loss, loneliness, and confusion. The effects of this were also evident in terms of the difficulties experienced by the young people in their own social relationships.

Studies described the negative effects of parental absence; this could be due either to parents separating (Forrest Keenan et al., 2007; Kjoelaas, Jensen, & Feragen, 2022) or the parent with HD becoming unavailable due to admission to hospital or a care home. The non‐HD parent was also sometimes unavailable due to their caring role or need to work (Forrest Keenan et al., 2007; Williams et al., 2009). This created difficulty in achieving a joint approach or joint understanding: ‘my mum (is) working two jobs…it's kind of hard for my dad to understand her stresses and it's hard for her to understand my dad's stresses…it just (kind of) creates an irritable mess’ (Sparbel et al., 2008, p. 331).

The absence of parents often left young people feeling alone and isolated with an intense sense of loss for both parents. This was often accompanied by the awareness of their parents' own losses: ‘my dad has been without a job ever since my mum got sick. So, in a way he got sick too. He doesn't seem to live any more either’ (Kjoelaas et al., 2020, p. 133).

Some young people shared their realization how, in retrospect, HD had stolen their parent for years before a diagnosis was given. Finding out about the presence of HD caused participants to question their childhood experiences and feelings towards their parents. This was particularly the case for young people whose HD diagnosed family member had been aggressive, violent or abusive and inconsistent in their boundaries, emotional responses and discipline (Forrest Keenan et al., 2007, 2009; Kjoelaas et al., 2020; Kjoelaas, Jensen, & Feragen, 2022; Mand et al., 2015). For example: ‘my dad is really passive and gentle and friendly, he's very calm, and never says a bad word about anyone, and all of a sudden he was belting my sister and belting the dog, really cutting himself off from the family, really detached, really angry’ (Mand et al., 2015, p. 211). Prior to knowing about HD, some participants were confused by their parents' behavior, often attributing it to being drunk (Duncan et al., 2007). Finding out about HD created turmoil, questioning whether their parent was hostile and aggressive because of HD or because of who they were prior to HD (Forrest Keenan et al., 2007; Mand et al., 2015).

For those who had knowledge of the parent's HD diagnosis, young people spoke about the difficulties of managing the disease progression. They faced repeated losses with the changes in the parent's personality, as HD seemed to become more dominant, rendering them unable to maintain their parent/child relationship (Kjoelaas et al., 2020; Kjoelaas, Jensen, & Feragen, 2022; Sparbel et al., 2008) or move on in their grief. There was an air of desperation in some reports of the need to grasp the last parts of life with the HD parent and value those moments together, before time ran out: ‘trying to, you know, enjoy the last poofs of my mum before something really bad happens’ (Williams et al., 2009, p. 282).

The sense of responsibility young people expressed in having to protect those around them, especially if the young person was a carer, was high (Forrest Keenan et al., 2009; Kjoelaas, Jensen, & Feragen, 2022). This sense of responsibility often resulted in young people leaving education and taking on typically adult roles in managing the home or becoming carers (Forrest Keenan et al., 2007; Sparbel et al., 2008). Young people also experienced not being able to sustain friendships and feeling misunderstood by friends when they chose caring over friendship (Kavanaugh et al., 2015). Some young people were too embarrassed or apprehensive to bring friends home (Kjoelaas et al., 2020) or reported that friends were avoidant of visiting (Sparbel et al., 2008), meaning that social relationships were difficult to both start and maintain.

Some young people tried to find positive ways to cope, such as keeping distance between the disease and their parents' identity which helped them make sense of difficult behavior and physical care needs. The refusal to place blame on their HD parent for their behavior was evident: ‘I know that she doesn't mean to, if she wasn't sick, she wouldn't be this way’ (Mand et al., 2015, p. 212).

Feelings were also not always negative as sometimes, despite the loss, participants viewed the non‐HD parent providing care with admiration and respect (Sparbel et al., 2008) and described them as a ‘hero’ (Forrest Keenan et al., 2009) or ‘lightening rod’ (Kjoelaas, Jensen, & Feragen, 2022) as they protected others from the impact of HD. This admiration seemed to be expressed when young people witnessed family members' difficult journey when caring for a partner with HD, suggesting the young people in the home were aware of what was occurring despite their relatively young age. Ultimately, growing up with an awareness of HD had a significant impact on all the young people's relationships.

### 3.2 Thief of self

HD stole the young person's sense of self. Within this theme is the impact of HD on a variety of areas in the young people's lives that removed aspects of their identity. This was stolen by HD triggering distressing thoughts about areas such as genetic status and testing, life choices, and decision‐making which seemed to have a negative impact on mental health and esteem. Young people found it difficult to identify and state what their needs were in the face of such dominant other family needs.

Living with inconsistent behavior from parents affected young people's understanding of themselves and their mental health, resulting in low self‐esteem and loss of security and stability (Kjoelaas, Jensen, & Feragen, 2022): ‘I became very insecure…unsure of what I had done wrong’ (Kjoelaas, Jensen, & Feragen, 2022, p. 217). Changes in parental boundaries (what was acceptable childhood behavior one day might not have been acceptable behavior the next) were described as ‘…an absurdity you cannot understand as a child’ (Kjoelaas et al., 2020, p. 133), resulting in anxiety and apprehension.

Caring roles of the child often extended past the immediate family home where other members of the family were HD positive (Forrest Keenan et al., 2007) such as supporting aunts, uncles, or cousins in a HD positive household; this required them to put themselves and their needs on a *‘back burner’* (Kavanaugh et al., 2015). In taking on this responsibility, the identity of the child was lost, with the child often taking a parent role in their relationships (Dondanville et al., 2019). No study documented that the child wanted to take on, or reject, such roles; rather, it was viewed as a duty to take care of the ones they loved (Dondanville et al., 2019).

Young people shared how being a child in the HD family felt as though they were unseen (Kavanaugh et al., 2015; Kjoelaas et al., 2020) and left without protection or provision (Kjoelaas, Jensen, & Feragen, 2022): ‘…the kids always seem to get ignored, I mean you were there but you're not listened to…’ (Forrest Keenan et al., 2009, p. 1895). Some explained how they protected themselves by becoming invisible: ‘I sort of erased myself…’ (Kjoelaas, Jensen, & Feragen, 2022, p. 219). Thus, young people were not acknowledged in their family homes amid the chaos of the disease. Despite this chaos, some participants were able to find a different part of their identity in sports, clubs, and with other relatives, which helped increase their sense of belonging, inclusion and being loved (Kjoelaas, Feragen, & Jensen, 2022), although this seemed rare.

Some young people wanted to know their genetic status as they believed this knowledge would give them control over their lives and a sense of a confirmed identity (Mand et al., 2015). Knowing that their test result was positive, and life could potentially be short, some participants described how they chose to live differently, engaging in more meaningful ways to spend their time and gaining a different perspective on life problems: ‘knowing that time is limited makes things that would otherwise seem like bigger deals really seem like not a deal at all’ (Gong et al., 2016, p. 1190). However, some also experienced distress upon hearing the test result, regardless of the outcome (Duncan et al., 2007).

Some young people at risk described themselves as living as though they were gene positive so that they self‐prepared for symptoms or a positive result if they were to get tested (Duncan et al., 2007; Forrest Keenan et al., 2015). Similarly to those who tested positive, they described self‐monitoring for symptoms (Forrest Keenan et al., 2007) and high levels of anxiety were reported if they noticed a trait in themselves that was like that of their family member with HD (Forrest Keenan et al., 2007). Living at risk was described as ‘… having a noose around your neck constantly. You don't know if it's there or not and that's what makes it so much harder. I have a 50/50 (chance of a) death sentence’ (Kjoelaas et al., 2020, p. 135). The impact on self of test‐taking was viewed differently by different participants; one participant expressed how growing up gene positive would be a *‘weird’* experience (Mand et al., 2015) but living at risk was somehow easier to understand and ‘mould yourself around’ (Mand et al., 2015, p. 213).

The management of the news of being gene positive was described as a process for some; the knowledge became less salient as time progressed ‘and then one day, it just goes to the back of your mind’ (Forrest Keenan et al., 2007, p. 125). However, for others, being gene positive influenced decisions not to have a family or take time investing in careers and instead they lived life with the motto of ‘work harder, achieve it faster’ (Gong et al., 2016, p. 1190), living within a window of allocated time. This expanded into decisions around partners and ensuring that, for those who wanted to have a family, they searched for an older partner with the priority of having children (Gong et al., 2016) rather than choosing a partner for other reasons. Some reported settling down with someone who knew about their genetic status through fear that no one else would accept them (Gong et al., 2016). Thus, HD continued to dictate life choices and family life.

### 3.3 Thief of transparency

HD stole transparency and instigated secret keeping. Within this theme is a lack of transparency concerning disclosure. Some families kept the presence of HD to themselves only allowing a select few to know about its existence. Being able to keep HD a secret also seemed to improve aspects of life for some young people such as protecting family and enabling young people to be the same as their peers.

Some families did not discuss HD with each other despite HD being present and family members being able to see the effects of the disease (Forrest Keenan et al., 2015; Kjoelaas et al., 2020; Mand et al., 2015). Keeping the presence of HD a secret in these ways resulted in the absence of internal familial and external social support (Forrest Keenan et al., 2015; Mand et al., 2015). Explanations for the secrecy were that families viewed HD as shameful (Mand et al., 2015), feared judgment from friends, were concerned about the impact on employment and insurance cover (Gong et al., 2016) or were unsure of when the right time would be to tell their children (Kjoelaas et al., 2020).

Some families did not speak about HD until the effects of the disease were becoming unavoidable, despite the young people in the home being aware of the stage of progression (Forrest Keenan et al., 2009). Then, sharing the news of HD and its effects came as a shock to the young people (Kjoelaas et al., 2020). Parents who were in denial about their own status and symptoms were reported to feed into the idea of keeping the disease a secret (Kjoelaas, Feragen, & Jensen, 2022). This had a negative effect on young people who wanted support or help but felt unable to pursue it: ‘it's the most difficult part about this whole thing when you are a young carer who wants help, but you are not getting anywhere because your parent is denying that they have a disease’ (Kjoelaas, Feragen, & Jensen, 2022, p. 665).

For those who did speak about HD, family support was not sought by some young people who expressed a preference to withhold their worries and concerns from family to protect them from the effects of their parents' genetic history (Dondanville et al., 2019; Kjoelaas, Feragen, & Jensen, 2022). However, young people who knew the family HD history and felt they had a family member who truly understood them and their experience felt close to that person (Forrest Keenan et al., 2009). The supporting person (e.g., non‐HD parent) could then interject and support the child during unreasonable challenges from the HD positive parent, indicating the supportive power of being transparent (Kjoelaas, Jensen, & Feragen, 2022).

Others shared how, within their immediate family, HD could be spoken about and discussed but outside of the family system it was not discussed or shared (Forrest Keenan et al., 2007; Kjoelaas et al., 2020; Mand et al., 2015). The opposite of this also occurred, with immediate family not discussing the disease but those closely linked, such as cousins, sharing the news and genetic risk of the disease with the young people (Duncan et al., 2007), resulting in negative familial impacts such as lack of trust. Despite how they were told, it appears that young people who were told about HD coped better with the impact of the disease and making life adjustments than those who were not told. Those living in families where HD was openly discussed had a strong awareness of how the disease shortened life expectancy for their parent and the possibility that HD could shorten their lives too. This brought an appreciation for life and awareness of how much time they might have left (Forrest Keenan et al., 2015).

In terms of how transparency affected social relationships, some young people reported why they kept HD a secret from their peers when they could: ‘I didn't want them to treat me differently. I just wanted to be the same as everyone else’ (Kjoelaas, Feragen, & Jensen, 2022, p. 668). Some young people had a desire to be able to exist as an individual outside of the disease, to be viewed in the same way as their peers and have a life that was independent of HD; this removed the ability of the young people to be able to choose to be transparent.

### 3.4 Search for reclamation

Young people wanted to reclaim aspects of their lives stolen from them. Within this theme, young people described trying to reclaim a sense of normality, searching for understanding and validation, information, and professional support in their quest for acceptance. For some, this search was successful at times, though it was hard and arduous for all.

Young people used varied methods to cope with their situation. One such method was an attempt to bring a sense of normality to their situation. Some young people held the view that they were a regular family despite HD and that they should be treated as such (Kavanaugh et al., 2015), which brought a sense of resilience and self‐preservation to their approach. Normalization of HD as a family illness or family problem or *‘quirk’* seemed to help families adjust and cope (Forrest Keenan et al., 2007, 2009; Kavanaugh et al., 2015).

Recognition of the difficulties experienced and support through these was desired but was not always available. Some young people wanted family and friends to accept that they could not understand the young person's situation but nevertheless could offer recognition of how difficult it was (Forrest Keenan et al., 2015; Gong et al., 2016; Kavanaugh et al., 2015; Kjoelaas, Feragen, and Jensen, 2022; Sparbel et al., 2008; Williams et al., 2009). However, others felt such recognition and support was impossible: ‘I don't think they understand that they don't have the full picture and that I don't feel like I can talk to them at all. Even though I know they are only trying to be supportive (and) they want me to talk to them and want to help me, it's like….it only makes it worse’… (Kjoelaas, Feragen, & Jensen, 2022, p. 666).

Some young people actively sought information for example, via the TV, internet, or through taking part in research, to find out about the disease and what was happening in their family (Forrest Keenan et al., 2009). Some reported being shocked to learn of their 50% risk (Forrest Keenan et al., 2007, 2009) having not realized the ‘family problem’ was a hereditary genetic condition. As well as aiding transparency (Theme 3), having a name to the problem and explanation of what was happening helped young people accept and deal with the disease and not see it as overwhelming (Forrest Keenan et al., 2009): ‘…the more information I got, the safer I felt. In terms of the possibility that I could become ill one day too, I learned that everyone with HD is not the same. That was really good to know.’ (Kjoelaas, Feragen, & Jensen, 2022, p. 664). This seemed to be in line with young people's experiences of professional support regarding gaining information.

Young people wanted support with finances and planning for future caring needs (Kavanaugh et al., 2015) but professional support was sparse (Forrest Keenan et al., 2009; Kjoelaas, Feragen, & Jensen, 2022; Williams et al., 2009). In particular, young people wanted support with the emotional aspects of their journey (Forrest Keenan et al., 2015), as this was not routinely provided by genetic counselors or other sources even after finding out their results (Gong et al., 2016). Professionals gaining a true understanding of the situation were described as a key factor for young people feeling supported and connected to others (Kjoelaas, Feragen, & Jensen, 2022). However, young people were also aware of negative effects some support could have. For example, they worried that a parent may be assessed as not able to provide safe care or needing additional professional care. Such fear often resulted in avoidance of support seeking.

Young people who had formed meaningful connections with another family member (aunt, uncle, and grandparent), a social group or a professional service seemed able to cope better due to the sense of connection and belonging such relationships gave them. This dynamic support was not available to most young people and to those who did have access to such support, many challenges were experienced. These challenges included the desire to self‐protect so that the young person did not feel overwhelmed, the desire to protect the family from intrusion and judgment, and the young person feeling misunderstood due to professionals' lack of HD knowledge and withdrawing from support as a result (Kjoelaas, Feragen, & Jensen, 2022).

Self‐acceptance was a key factor in being able to move on and hold HD in a place in the mind that enabled people to continue with life (Dondanville et al., 2019; Duncan et al., 2007; Forrest Keenan et al., 2007). Having a positive attitude and making plans (Dondanville et al., 2019; Forrest Keenan et al., 2007), as well as finding out their own HD status and a sense of hope, aided them taking control of life (Duncan et al., 2007) and feeling more able to adjust, cope, and make decisions.

## 4 DISCUSSION

This meta‐ethnography found that growing up in a family with HD can be a multifaceted and challenging experience. The complex interplay between the loss of relationships, sense of self, and the ability to be open and transparent often resulted in susceptibility to mental health challenges such as low mood, low confidence, low esteem, and feelings of isolation. In efforts to reclaim what was stolen and control some of the impact on their lives, young people had to face their fears and their own feelings about themselves, worries about their family members, fears of becoming like their parent and what that might mean for them. Young people sought support; however, this was sparse and inconsistent in quality and availability.

In Theme 1 (thief of relationships), young people expressed how the unavailability of their parents was difficult to manage. Such absence may have influenced parent/child attachment. Attachment theory (Bowlby, 1979) argues that it is important that parents are attuned to their children's needs (Howe, 2011) for children to develop secure, safe, and meaningful relationships. Van der Meer et al. (2006) found that HD families, including young people with a HD parent, experienced higher levels of preoccupied and unresolved/disorganized attachments and lower levels of secure attachment styles when compared to a non‐HD population. The lack of a secure parent figure or secure base providing comfort and support could result in the young people developing a negative attachment style (Bowlby, 1988) which could cause difficulty relating to the self and other people resulting in psychological and emotional distress that extends into future relationships (McCarthy & Taylor, 1999).

Social relationships were also affected, with young people feeling unable to let peers fully into their lives. Having social connections with peers and communities is important as people with such connections are generally happier, have better physical and mental health, and live longer (Holt‐Lunstad et al., 2010). Peer‐to‐peer relationships form an essential part of development concerning cognition, social interaction (Rubin et al., 2011), prosocial behaviors (Eisenberg et al., 2019), emotional regulation and adjustment (Contreras & Kerns, 2000), and reciprocal roles, which are essential in forming healthy relationships and a sense of identity and belonging (Parker et al., 2006).

Theme 2 (thief of self) showed how HD interfered with the identity of young people and negatively affected their mental health. Adverse childhood experiences (ACEs) have been associated with psychological distress and poor physical health outcomes in non‐HD populations (e.g., Hughes et al., 2017). Young people in HD families have often experienced multiple ACEs (e.g., anticipatory mourning, loss of a member of their family unit, possible domestic violence and aggression in their home) placing them at higher risk of future physical and psychological health complications (Monnat & Chandler, 2015). As ACEs originate mostly, though not always, from parent–child relationship disruptions, young people in HD families may be at risk of developing a negative view of the self and poor social skills resulting in compromised identity development (Wong et al., 2019).

Another finding of this review is the negative effect of secrecy (Theme 3: thief of transparency). A study with young people living with chronic illness found the main factor influencing illness disclosure with peers was that the peer had a shared experience with illness (Kaushansky et al., 2017). When disclosure did not occur, similarly to the young people in this review, this was due to fear of rejection, pity, or being viewed as different (Kaushansky et al., 2017). HD research with adults documents various examples of secrecy such as hidden coping strategies, including substance misuse (Aubeeluck & Moskowitz, 2008), recognizing but disguising personality or health changes in family members (Hayes, 1992), the bidirectional nature of secret keeping between the member with HD, spouse and other family members (Kessler, 1993) and the impact of secret keeping (Forrest Keenan et al., 2013). These examples were also identified within this review which focused solely on young people.

Despite the challenges that living in a family with HD brought, young people fought to live their own lives (theme 4: search for reclamation). One way reclamation was sought was through acceptance of HD as being a normal part of family life. Studies on chronic illness document the positive effect of acceptance on a person's ability to adapt to living with illness (Livneh et al., 2006; Telford et al., 2006). Other studies have also noted that in families where HD was openly discussed and the presence of the genetic risk and disease accepted, positive changes were reported such as a change in perspective and an ability to focus on enjoyable experiences (Maxted et al., 2014).

### 4.1 Clinical implications

Within theme four, young people spoke about the importance of feeling understood, both by their friends and family, but also by professionals. While information provision was valued, emotional support was frequently highlighted as lacking. Well placed to meet this need are professionals such as psychologists and mental health specialists although access is internationally recognized as problematic.

When providing support to young people and their families, both the systems surrounding a young person, and the young person as an individual, need to be considered. Biopsychosocial models adapted for families with genetic illnesses, such as the Family Systems Genetic Illness Model (Rolland & Williams, 2005), suggest a family focused method to enable effective communication of genetic risk and then understand the impact of such risk on the family unit (Miller et al., 2006).

A resilience framework, such as the strength‐based cognitive therapy four step model (Padesky & Mooney, 2012), may also be useful to explore with young people in HD families and may add a preventative aspect. Such a model focuses on strengths and brings these into awareness using imagery and metaphor. The goals of this are to increase resilience rather than reach resolution. While such an individual‐focused approach may be useful, there is also value in exploring family‐focused interventions that may assist young people.

Future research could include exploring ways of improving the assessment and formulation of young people in HD families, and the need for psychological support. A recent formulation model for use with individuals affected by HD has been proposed (Dale et al., 2022). The model offers prompts for individual components that require consideration within the lives of those affected by HD (e.g., HD narrative, HD triad of symptoms) and offers guidance for professionals to facilitate an effective HD formulation. This could be adapted for young people in HD families to provide guidance for professionals with little knowledge of HD concerning what areas need to be discussed.

This review also highlights that most research on young people and HD draws out the negative aspects of living in a HD family. It may be beneficial to focus on the positives that young people can experience, adopting a more positive psychology perspective (Seligman & Csikszentmihalyi, 2000) to understand further the life experience of this group and progress away from an overly pathological‐laden narrative (Barak & Achiron, 2009).

### 4.2 Limitations

Although 13 papers were included, these represent only 9 studies, and this may have impacted the representation of second order constructs. All studies were conducted in western countries and, therefore, the review does not represent the experiences of young people who do not have access to healthcare and support systems present in emerging economies. The CASP tool revealed that few papers described the researchers' possible influence on data analysis; therefore, future research may benefit from explicit statements concerning reflexivity. Lack of reflexivity means the relationship between the participants and researchers is not made clear in some papers, which could mean that their findings are biased in some way that is hard to assess. However, given that a meta‐ethnography synthesizes findings across studies, the impact on the findings as a whole may be reduced somewhat. The lack of explicit consideration of ethical issues in the papers is concerning, but may also be an effect of limited journal word counts. There was no evidence of unethical practice, and a paper would not have been included if this were the case. If participants had misunderstood the aims of the research or felt pressurized to take part (part of the CASP assessment of ethical practice), it is possible the data collected would not be valid, but without evidence of this, it is hard to assess any impact on the data.

## 5 CONCLUSION

The review highlights the significant level of distress experienced by young people in families with HD. Similarly, quantitative studies have reported that young people in HD families experience high levels of emotional, social, and practical burden (Lewit‐Mendes et al., 2018) and that HD families experience high levels of dysfunction, including sub‐optimal parenting (control and abuse) from both the HD and non‐HD parent (Vamos et al., 2007). The qualitative approach of this review adds the experiential voice of young people and a detailed understanding of the contributing factors that have resulted in such distress through the descriptions of HD as a thief and young people's resulting need to try and reclaim aspects of themselves and their lives.

## AUTHOR CONTRIBUTIONS

Authors Dr Hollie Cooper, Professor Jane Simpson, Dr Maria Dale, and Dr Fiona Eccles contributed to this work. Hollie Cooper made substantial contributions to the acquisition, analysis, and interpretation of data for the work which was supported by Professor Jane Simpson, Dr Maria Dale, and Dr Fiona Eccles. All authors made substantial contributions drafting the work and revising it critically for important intellectual content.

## CONFLICT OF INTEREST STATEMENT

Authors Dr Hollie Cooper, Professor Jane Simpson, Dr Maria Dale, and Dr Fiona J.R. Eccles declare that they have no conflict of interest.

## Supporting information


Tables S1–S3
