# Supplementary material for: Experiences of young people growing up in a family with Huntington's disease: A meta‐ethnography of qualitative research
Source: J Genet Couns. 2024 Mar 12;34(1):e1886. doi: 10.1002/jgc4.1886 (PMC11726609; doi:10.1002/jgc4.1886)
Supplement: Supplementary file 1 — Tables S1–S3 [file JGC4-34-0-s001.docx]

Supplementary Files

**Table 1**

*Systematic Review Search Strategy example (PsycINFO)*

| 1 (qualitative research) | ( ^^[[1]](#footnote-1)^^DE "Qualitative Measures" OR DE "Questionnaires" OR DE "General Health Questionnaire" OR DE "Interviewing" OR DE "Interviewers" OR DE "Attitudes" OR DE "Abortion (Attitudes Toward)" OR DE "Adolescent Attitudes" OR DE "Adult Attitudes" OR DE "Aged (Attitudes Toward)" OR DE "Aging (Attitudes Toward)" OR DE "Attitude Change" OR DE "Attitude Formation" OR DE "Attitude Similarity" OR DE "Child Attitudes" OR DE "Childrearing Attitudes" OR DE "Client Attitudes" OR DE "Community Attitudes" OR DE "Computer Attitudes" OR DE "Consumer Attitudes" OR DE "Counselor Attitudes" OR DE "Cultural Attitudes" OR DE "Death Attitudes" OR DE "Disabled (Attitudes Toward)" OR DE "Drug Usage Attitudes" OR DE "Eating Attitudes" OR DE "Educational Employee Attitudes" OR DE "Employee Attitudes" OR DE "Employer Attitudes" OR DE "Environmental Attitudes" OR DE "Explicit Attitudes" OR DE "Family Planning Attitudes" OR DE "Female Attitudes" OR DE "Gender Role Attitudes" OR DE "Health Attitudes" OR DE "Health Personnel Attitudes" OR DE "Ideology" OR DE "Implicit Attitudes" OR DE "Job Applicant Attitudes" OR DE "Law Enforcement Employee Attitudes" OR DE "Male Attitudes" OR DE "Marriage Attitudes" OR DE "Obesity (Attitudes Toward)" OR DE "Occupational Attitudes" OR DE "Parental Attitudes" OR DE "Paternalism" OR DE "Political Attitudes" OR DE "Preferences" OR DE "Psychologist Attitudes" OR DE "Public Opinion" OR DE "Racial and Ethnic Attitudes" OR DE "Sex Role Attitudes" OR DE "Sexual Attitudes" OR DE "Socioeconomic Class Attitudes" OR DE "Sports (Attitudes Toward)" OR DE "Stereotyped Attitudes" OR DE "Student Attitudes" OR DE "Teacher Attitudes" OR DE "Work (Attitudes Toward)" OR DE "World View" OR DE "Ethnology" OR DE "Phenomenology" OR DE "Discourse Analysis" OR DE "Observation Methods" OR DE "Direct Observation" OR DE "Participant Observation" OR DE "Qualitative Methods" OR DE "Focus Group" OR DE "Grounded Theory" OR DE "Interpretative Phenomenological Analysis" OR DE "Narrative Analysis" OR DE "Semi-Structured Interview" OR DE "Thematic Analysis" ) OR TI ( mixedmethod* OR "mixed method*" OR mixed-method* OR qualitative OR interview* OR experience* OR "focus group∗" OR ethnograph∗ OR fieldwork OR “field work” OR “key informant” OR ((“semi-structured” OR semistructured OR unstructured OR informal OR “in-depth” OR indepth OR “face-to-face” OR structured OR guide) N3 (interview∗ OR discussion∗ OR questionnaire∗)) ) OR AB ( mixedmethod* OR "mixed method*" OR mixed-method* OR qualitative OR interview* OR experience* OR "focus group∗" OR ethnograph∗ OR fieldwork OR “field work” OR “key informant” OR ((“semi-structured” OR semistructured OR unstructured OR informal OR “in-depth” OR indepth OR “face-to-face” OR structured OR guide) N3 (interview∗ OR discussion∗ OR questionnaire∗)) ) |
| --- | --- |
| 2 (Huntington’s Disease) | DE "Huntingtons Disease" OR TI huntington* OR AB huntingto |
| 3 | #1 AND #2 |

**Table 2**

*Critical Appraisal Skills Programme*

| CASP | Dondanville et al., (2018) | Duncan et al., (2007) | Forrest-Keenan et al., (2007) | Forrest Keenan et al., (2009) | Forrest Keenan et al., (2015) | Gong et al., (2016) | Kavanaugh et al., (2015) | Kjoelaas et al., (2022a) | Kjoelaas et al., (2022b) | Kjoelaas et al., (2020) | Mand et al., (2015) | Sparbel et al., (2008) | Williams et al., (2009) |
| --- | --- | --- | --- | --- | --- | --- | --- | --- | --- | --- | --- | --- | --- |
| Was there a clear statement of the aims of the research? | Y | Y | Y | Y | Y | Y | Y | Y | Y | Y | Y | Y | Y |
| Is qualitative methodology appropriate? | Y | Y | Y | Y | Y | Y | Y | Y | Y | Y | Y | Y | Y |
| Was the research design appropriate to address the aims of the research? | U | U | Y | Y | Y | Y | Y | Y | Y | Y | U | Y | Y |
| Was the recruitment strategy appropriate to the aims of the research? | Y | Y | Y | Y | Y | Y | Y | Y | Y | Y | U | Y | Y |
| Was the data collected in a way that addressed the research issue? | Y | Y | Y | Y | Y | Y | Y | Y | Y | Y | Y | Y | Y |
| Has the relationship between researchers and participants been adequately considered | N | U | U | U | U | Y | U | Y | U | U | N | U | U |
| Have ethical issues been taken into consideration? | U | U | U | U | U | U | U | Y | Y | Y | U | Y | Y |
| Was the data analysis sufficiently rigorous? | U | Y | Y | Y | Y | Y | Y | Y | Y | Y | U | Y | Y |
| Is there a clear statement of findings? | Y | Y | Y | Y | Y | Y | Y | Y | Y | Y | Y | Y | Y |
| How valuable is the research? | Y | Y | Y | Y | Y | Y | Y | Y | Y | Y | Y | Y | Y |

Y – yes: the study fulfilled majority of the prompts per question

N – no: the study did not fulfil many of the prompts per question

U – unclear: difficult to establish a definitive yes or no in response to prompts per question

**Table 3**

*Table to illustrate constructs in included papers.*

|  | Dondanville et al., (2018) | Duncan et al., (2007) | Forrest Keenan et al., (2007) | Forrest Keenan et al., (2009) | Forrest Keenan et al., (2015) | Gong et al., (2016) | Kavanaugh et al., (2015) | Kjoelaas et al., (2020) | Kjoelaas et al., (2022a) | Kjoelaas et al., (2022b) | Mand et al., (2015) | Sparbel et al., (2008) | Williams et al., (2009) |
| --- | --- | --- | --- | --- | --- | --- | --- | --- | --- | --- | --- | --- | --- |
| Person vs HD | X | X | X | X | X | X | NA | X | X | NA | X | X | X |
| Relationships & connection | X | X | X | X | X | X | X | X | X | X | X | X | X |
| Relationships removed by HD | X | X | X | X | X |  | X | X | X | X | X | X | X |
| Education | X | X | X | X | X | X | NA | X | NA | NA | X | X | X |
| Childhood | X | X | X | X | X | X | X | X | X | X | X | X | X |
| Mental health | X | X | X | X | X | X | X | X | X | X | X | X | X |
| Identity | X | X | X | X | X | X | X | X | X | X | X | X | X |
| Choice/control | X | X | X | X | X | X | X | X | X | X | X | X | X |
| Age of testing, age to know about HD | X | NA | X | X | NA | X | NA | X | NA | NA | X | NA | NA |
| Understanding & information seeking | X | X | X | X | X | X | X | X | X | X | X | X | X |
| Professionals | X | X | X | X | X | X | X | X | X | X | X | X | X |
| Keeping secrets, denial | NA | X | X | X | X | X | NA | X | NA | X | X | NA | NA |
| Coping | X | X | X | X | X | X | NA | X | X | X | X | X | X |

1. DE refers to descriptors. These are specific subject terms that control how subjects are searched and have been created by the American Psychological Association. [↑](#footnote-ref-1)
